# Metagenome-assembled genome of *Staphylococcus nepalensis* from urban bats in China

**DOI:** 10.1128/mra.01222-24

**Published:** 2024-12-31

**Authors:** Xuan Jiang, Wentai Ma

**Affiliations:** 1 China National Center for Bioinformation, Beijing, China; 2 Beijing Institute of Genomics, Chinese Academy of Sciences, Beijing, China; 3 University of Chinese Academy of Sciences, Beijing, China; Montana State University, Bozeman, Montana, USA

**Keywords:** *Staphylococcus nepalensis*, *Pipistrellus abramus*

## Abstract

We conducted metagenomic sequencing on a stool sample collected from urban-dwelling *Pipistrellus abramus* and obtained a metagenome-assembled genome of *Staphylococcus nepalensis*. Phylogenetic analysis revealed that this strain is closely related to the one isolated from dogs, forming a distinct clade separate from the reference genome.

## ANNOUNCEMENT

Bats are natural hosts for many potential zoonotic pathogens (1, 2). While significant research has focused on bat populations in wild and rural areas (3), less attention has been given to urban areas where people have coexisted with bats.

We collected a stool sample from the windowsill of a downtown apartment (seventh floor) in Wuhu, Anhui, China, where bats have been observed. DNA was extracted from 200 mg of the sample using the DNeasy PowerSoil Pro kit (Qiagen, Germany). Library preparation was conducted using the Watchmaker DNA Library Prep Kit with Fragmentation (Watchmaker Genomics, USA), which enzymatically digested the DNA into 350 bp fragments without fragment selection following library amplification. Sequencing was performed on the DNBSEQ-T7 V3.0 platform, generating 99.89 million 150 bp paired-end reads.

Raw reads were filtered using fastp version 0.23.4 (4) and mapped to a eukaryotic mitochondrial database (5) using Minimap2 version 2.17 (6) to determine the bat species. Of the mapped reads, 7,980 (94.9%) matched *Pipistrellus abramus* (Japanese house bat), consistent with the observed bat’s characteristics (small, black-brown fur, rounded ears, and a long-enveloped tail). Since this species lacks a complete genome, we downloaded all four reference genomes from the genus *Pipistrellus* (GCA_903992545.1, GCA_949987585.1, GCA_963693515.1, and GCA_014108245.1) for host genome depletion with Bowtie2 version 2.5.4 (7). The resulting 48.86 million clean reads were classified using Kraken2 version 2.1.3 with its standard Refseq database (8). Among them, 43.01% were assigned to the genus *Staphylococcus,* while 39.56% were directly assigned to *Staphylococcus nepalensis*, making it the most abundant species. *S. nepalensis* is a bacterium commonly detected in insects, animals, and foods, and capable of causing infections (9–11).

*De novo* assemblies were generated using MEGAHIT version 1.2.9 (12). Contigs longer than 500 bp were retained and mapped to the reference (GCA_002442935.1) as long reads using Minimap2. To minimize ambiguity caused by homologous regions between species, each contig was amplified based on its average depth (provided by MEGAHIT) to restore its relative abundance before mapping. To fill uncovered regions between contigs, clean reads assigned to and under the genus *Staphylococcus* were extracted using SeqTK version 1.3 (13) and mapped to these gaps with Minimap2. The final consensus genome (Bat2024WH) was generated using VarScan version 2.4.6 and our custom script (14), with a minimum depth of 5 and a major allele frequency of 50% (otherwise the base is recorded as “N”), preserving all inDels. The assembly quality was evaluated using QUAST version 5.2.0 (15) and NCBI FCS version 0.5.4 (16). Software details are provided in Table 1.

**TABLE 1:** Bioinformatics software used in this study

Software	Version	Purpose	Parameters
Fastp	0.23.4	Remove adapters, polyX sequences, low-quality tails, and reads shorter than 50	-l 50 -x --detect_adapter_for_pe --cut_tail --cut_tail_mean_quality 20
Minimap2	2.17	Reads and contigs mapping	-x sr (for short reads) Default (for long reads)
Bowtie2	2.5.4	Host genome depletion	Default
Kraken2	2.1.3	Taxonomy classification	Default
MEGAHIT	1.2.9	*De novo* assembling	Default
SeqTK	1.3	Reads extraction	Default
VarScan	2.4.6	Generate readcounts file	readcounts --min-coverage 0
C ++ Script	0.1.1	Generate consensus sequence	-m 5 -t 0.5
QUAST	5.2.0	Genome assembly QC	Default
NCBI FCS	0.5.4	Genome assembly QC	Default
MAFFT	7.453	Multiple sequence alignment	--auto
MEGA X	10.1.8	Phylogenetic reconstruction	Default
iTOL	6.9.1	Tree visualization	Default

**Fig 1:**
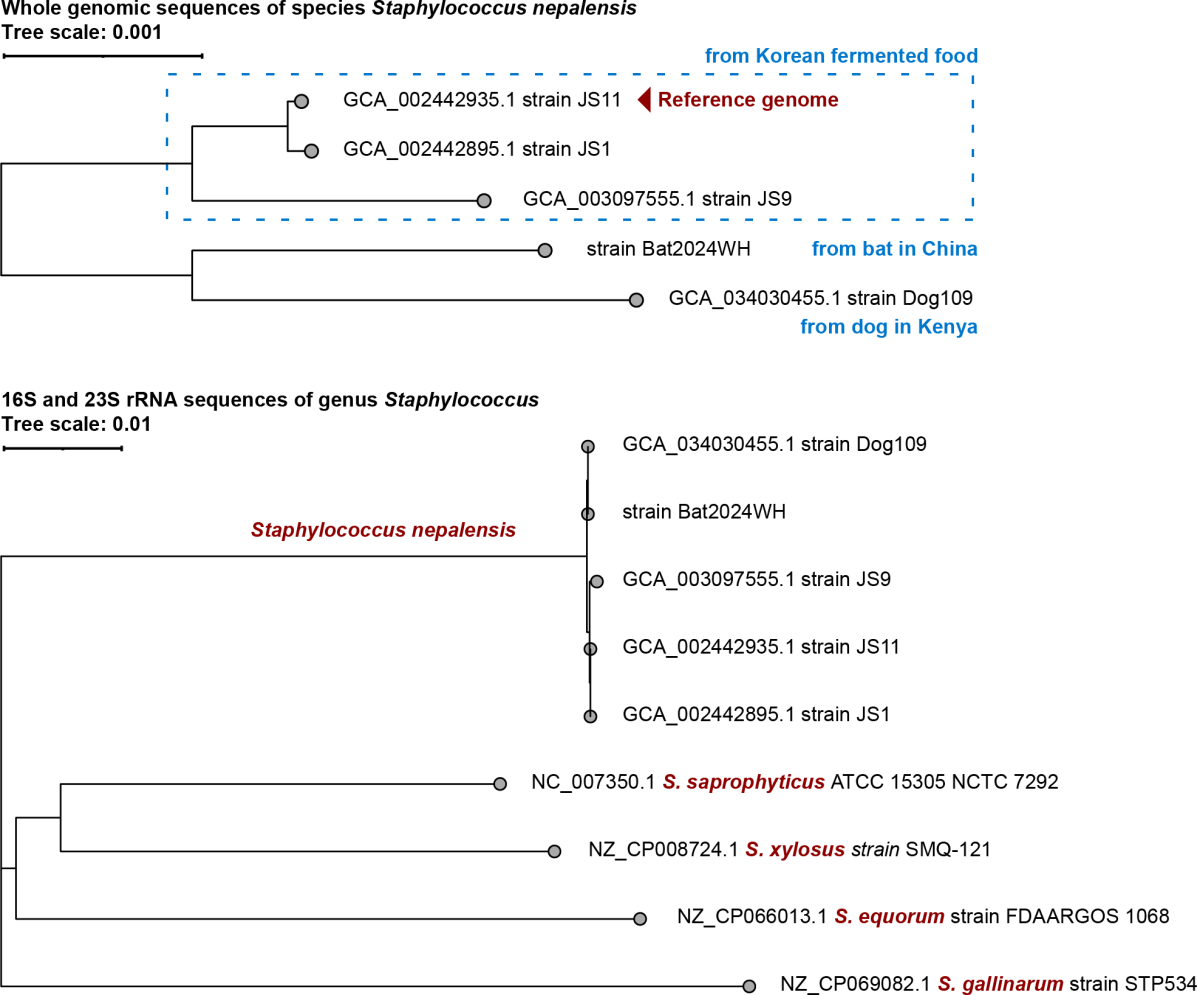
Phylogeny of the assembled genome Bat2024WH and other strains of genus *Staphylococcus*. The alignment was performed using MAFFT version 7.453 (17), and phylogenetic reconstruction was performed with MEGA X version 10.1.8 (18) using the neighbor-joining method. The tree was visualized using iTOL version 6.9.1 (19).

The Bat2024WH metagenome-assembled genome spans 2,837,036 bp, with a mean depth of 2,005.91× (96.96% of sites > 100×). Only 27,238 bp (0.96%) of the genome were covered by N. Phylogenetic analysis demonstrated that this strain is closely related to the one isolated from dogs, forming a distinct clade separate from the current reference genome isolated from fermented food (Fig. 1).

## Data Availability

This project is deposited in NCBI under accession number PRJNA1178833, with raw data available via SRA accession SRR31145840. The assembled MAG sequence is available in GenBank with accession GCA_044740705.1. The scripts and detailed pipeline are uploaded to GitHub (20).
